# An Approach for Fall Prediction Based on Kinematics of Body Key Points Using LSTM

**DOI:** 10.3390/ijerph192113762

**Published:** 2022-10-22

**Authors:** Bahareh Mobasheri, Seyed Reza Kamel Tabbakh, Yahya Forghani

**Affiliations:** Department of Computer Engineering, Mashhad Branch, Islamic Azad University, Mashhad 9187147578, Iran

**Keywords:** falls prediction, older adults, health promotion, wellness, LSTM, image processing

## Abstract

Many studies have used sensors attached to adults in order to collect signals by which one can carry out analyses to predict falls. In addition, there are research studies in which videos and photographs were used to extract and analyze body posture and body kinematics. The present study proposes an integrated approach consisting of body kinematics and machine learning. The model data consist of video recordings collected in the UP-Fall Detection dataset experiment. Three models based on long-short-term memory (LSTM) network—4p-SAFE, 5p-SAFE, and 6p-SAFE for four, five, and six parameters—were developed in this work. The parameters needed for these models consist of some coordinates and angles extracted from videos. These models are easy to apply to the sequential images collected by ordinary cameras, which are installed everywhere, especially on aged-care premises. The accuracy of predictions was as good as 98%. Finally, the authors discuss that, by applying these models, the health and wellness of adults and elderlies will be considerably promoted.

## 1. Introduction

Falls are common external causes of unintentional injuries. In everyday life, people might experience falls due to different reasons: illness, frailty, or loss of balance in any normal activity. Given that frequency of falls increases with age, older adults are more likely to experience falls than individuals in other age groups(Rajagopalan et al. [1]). Approximately 28–35% of people aged 65 and over fall each year, and this increases to 32–42% for those over 70 years of age (Habib et al. [2]). Failure to provide immediate care to the person who has fallen may lead to irreparable harm, such as chronic disability or death. Real-time detection and prediction of falls can help to reduce the negative consequences of falls as it allows for timely prevention and/or receiving medical attention. In such cases, having a fall detection system would be of great help.

There are three main techniques by which fall data are collected: ambiance sensors, wearable devices, and vision-based devices. The method of data collection is different in each of the mentioned techniques. Each of these techniques will be explained below.

Ambient sensors are sensors installed in the environment, such as infrared devices, floor-based devices, pressure sensors, etc. The data collected through ambient sensors are mostly used in activity detection models where the environmental conditions may affect correct detection. This study does not refer to work conducted using ambient sensors because the focus of this research, regardless of environmental conditions, is only on examining changes in key points of the body during a fall.

In wearable devices, data are frequently collected through inertial measurement units (IMUs), such as accelerometers and gyroscopes attached to different parts of the person’s body. This includes sensors embedded in smartphones, smartwatches, and other portable devices. Wearable-sensor fall detection approaches [3,4,5,6] use IMUs to characterize and detect a fall using time series data. They investigated a variety of deep learning (DL) models, such as LSTM, convolutional neural network (CNN), autoencoder-CNN-LSTM, decision tree (DT), K-nearest neighbor (KNN), and support vector machine (SVM) to detect falls. Another type of wearable-sensor-based approach for fall detection was reported by Shi and Chen [7]. They reported accurate fall prediction by applying a CNN technique and a class activation mapping (CAM) method. This highlighted the class-specific region in the data and obtained a hot map of the fall data. Learning was again based on the real-time data from an IMU-sensor mounted on the person’s waist.

Wearable devices have gained noticeable popularity among all other fall detection systems due to their ease of use and portability. However, the use of wearable sensors may have some flaws. Sensors may not always provide accurate data. Namely, the sensors that are measuring a person’s acceleration or rotation might show different measurements even if they are manufactured in the same company and have the same brand. Furthermore, the presence of noise in such data is inevitable (Marsico et al. [8]). Therefore, some preprocessing methods are needed to be applied to data to make them ready to be used in machine learning (ML) models, and this may be time-consuming. Another consideration is regarding misalignment of sensors that could lead to erroneous readings. There are also concerns about sensors’ battery replacement/charging; otherwise, they may have erroneous measurements. Since the target community of this research is mostly older adults and/or people who are unable to maintain their balance, it seems that attaching an external object, such as a sensor to their body, might cause inconveniences. 

However, use of data generated by video images does not seem to have the above-mentioned problems. Here are some of the newest methods in which video images are deployed for fall detection.

According to computer-vision fall detection methods [9,10,11], to distinguish between fall and non-fall events, the human head/body is tracked in consecutive frames using the best-fit approximated ellipse and projection histograms. In other studies [12,13,14,15], based on computer-vision, to distinguish falls from not-fall activities, the histogram of oriented gradient (HOG) and optical flow techniques were used to produce a human body’s contour. In order to implement their works, the authors in Refs. [12,13,14,15] have used a variety of ML and DL models, such as SVM, KNN, multi-layer perceptron (MLP), and CNN. Another computer-vision-based solution [16,17,18] for fall detection leverages the human skeleton from Kinect and OpenPose to obtain body key points. These key points are used to identify and predict falls using DL models, such as CNN, recurrent neural network (RNN), and LSTM. Compared to human contour recognition, skeleton recognition is less susceptible to environmental disturbance and offers better privacy protection. The training time is reduced since the training data are based on joint points rather than images. 

Finally, it seems that the fall prediction methods obtain more accurate results as they are more convenient to integrate image-processing techniques and machine-learning algorithms for reliable estimation of fall risks and provide timely alerts before the occurrence of a fall (Lin et al. [18]).

In our study, a motion description model based on body kinematic changes is established by detecting the human body in an active frame and marking the body’s skeleton key points. The fall behavior detection models proposed in this work are evaluated using well-known statistical parameters: precision, recall, F1-score, and accuracy.

This study aims to assist caregivers to predict falls in a timely manner using images extracted from videos recorded by surveillance systems and can play a significant role in intelligent safety systems for older adults. However, to be able to accurately differentiate between a fall and not-fall behavior, development of an intelligent system is needed. Such an intelligent system is fed by images and will be able to improve itself using feedback in order to achieve higher accuracies. 

The present study proposes *a System for Alarming of Fall in oldEr adults (SAFE),* an integrated approach consisting of body kinematics and ML techniques. The model data consist of video recordings collected in the UP-Fall Detection dataset experiment.

**Figure 1:**
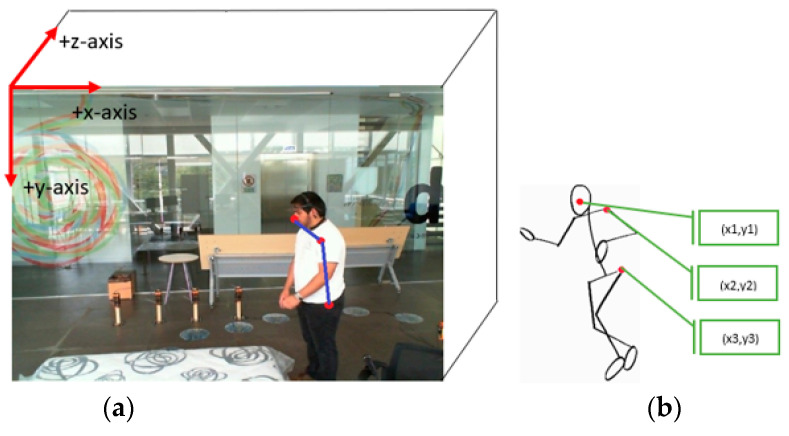
(**a**) A general view of the room where the experiments were conducted and the videos were recorded (Mardinez and Ponse [14]); (**b**) a simple skeleton shape indicating the points of interest on the subject’s body.

In this article, our contributions are:

- Using the MediaPipe framework algorithm (Algorithm 1), a simplified descriptive geometry using only three human skeleton key points is drawn (Figure 1). These key points could conveniently explore the characteristics of the human motion pose. Extracting only three body key points from the image, in addition to spending a short time for image processing, achieves high accuracy in fall detection.
- The output of the MediaPipe algorithm is used to calculate some angles for the selected three key points (Section 2.3.3). To the best of the authors’ knowledge, this is the first time that this approach has been used to detect falls.

## 2. Materials and Methods

### 2.1. Data and Materials

The data used in this work are the well-known UP-Fall Detection dataset (Mardinez and Ponse [14]). This dataset consists of falls and several activity daily living (ADL) data. The data are acquired by a multimodal approach using 14 devices to measure activity in three different ways using wearables, context-aware sensors, and cameras, all at the same time. Data were collected from 17 healthy young adults called subjects (9 male and 8 female) ranging from 18 to 24 years of age without impairment. The subjects’ mean height was 1.66 m, and their mean weight was 66.8 kg. Of course, collecting these data for older adults is more desirable, but the risk of such a simulation is too high because of possible injuries and harm. On the other hand, since we are looking for body posture of some key points, angles, and coordinates, the age of subjects may have little influence on the results. 

Wearable sensors consisted of inertial sensors collecting raw data, namely 3-axis accelerometers, 3-axis gyroscopes, and the ambient light sensors. These wearables sensors were located on the left wrist, on the neck, in the right pocket of their pants, in the middle of the waist (on the belt), and on the left ankle. Moreover, one electroencephalogram (EEG) headset was worn by all subjects on their foreheads in order to measure the raw brainwave signal. The ambient sensors used were six infrared sensors as a grid. The infrared sensors measured the changes in the interruption of the optical devices and were installed 0.40 m above the floor. Keeping in mind that the focus of this research is only on examining changes in key points of the body during a fall, we only use images received from video cameras and, therefore, do not refer to data gathered using wearable sensors or any of the context-aware sensors, such as EEG, and ambient sensors.

The cameras used were Microsoft LifeCam Cinema cameras, which were located 1.82 m above the floor. Cameras were used to capture video images of subjects, with a resolution of 640 × 480 pixels. Along with these images, the time-stamp label and the target tag (Activity ID) in each image were also stored in the dataset. Two cameras were used for this task, one for a lateral view and the other for a frontal view. The sampling frequency of images was tuned at ∼18 Hz and in a time-series order.

The UP-Fall Detection dataset comprises 11 activities, including 6 human daily activities (i.e., walking, standing, picking up an object, sitting, jumping, and lying down) and 5 human falls (falling forward using hands, falling forward using knees, falling backward, falling while sitting in an empty chair, and falling sideward). Three trials are recorded for each activity. Since this work is based on the information extracted from frame images, only images acquired by the lateral camera were used. Given that the purpose of this study is to find a new approach to detecting falls, only images of subjects experiencing forward and sideward falling were deployed. Note that the UP-Fall Detection dataset has a time label.

### 2.2. System Configuration

Processing and calculations were completed at the TUF Gaming FX505DV_FX505DV workstation based on an NVIDIA GeForce RTX 2060, an AMD Ryzen 7 3750H with Radeon Vega Mobile Gfx, 4 cores, and 64 GB memory. On the software side, Python (version 3.8.5) was used to code the model in a Jupyter notebook (version 2.2.6). The preprocessing, preparation, and training of the models were completed in Python. The final model was set up with Tensorflow (API 2.5.0). Loading and managing the data were completed in the pandas (version 1.1.3) and NumPy (version 1.19.5) libraries. Matplotlib (version 3.3.2) was used for visualization of the data.

### 2.3. Methodology

To diagnose falling, the points on the body that have the greatest impact on maintaining subject stability were used. The points that are commonly selected in similar research are the nose, shoulders, hips, and knees. Finally, the data used in this research are the coordinates of 3 key points of the body in the three directions of X, Y, and Z and are extracted starting from frames where the subject is still in a stable condition. In the first stage of image processing, the coordinates of specified body key points were extracted from each image. 

The parameters primarily considered for fall detection consisted of:

- Coordinates of the nose, left shoulder, right shoulder, left hip, right hip, left knee, right knee.
- The rate of change in the angle between the connected line from the nose, left shoulder, right shoulder, left hip, and right hip body key points to the origin with the x-axis.
- The ratio of the y-coordinate of nose to the y-coordinate of left hip in two consecutive frames.

**Figure 2:**
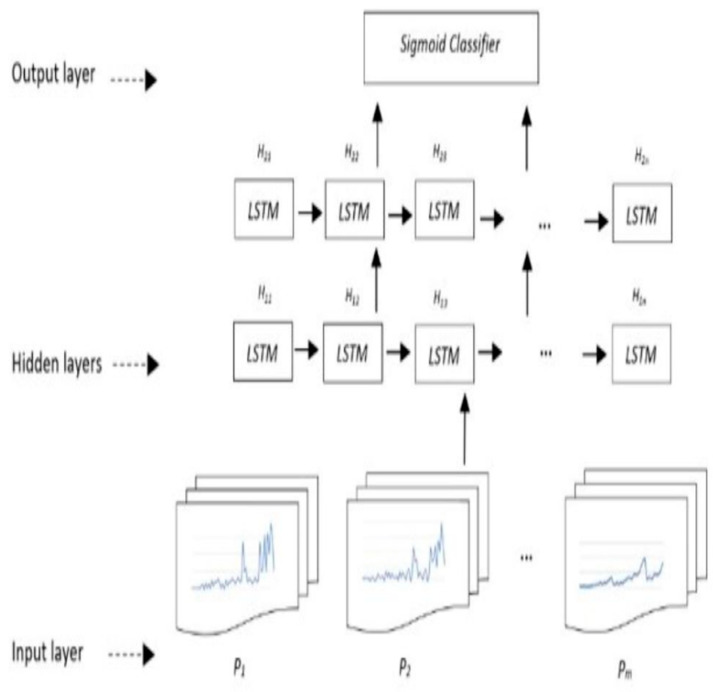
An overview of the LSTM network used in this study.

The performance of each one of these parameters was investigated, where the best of them were used in this work. However, too many feature parameters may cause the recognition rate to either decrease or become more complicated. By testing many combinations of different parameters, only 3 points were retained as training feature values. As shown in Figure 2, the body key points used in this work were: nose (N), left shoulder (LSh), and left hip (LH). 

With extraction of the body key points in each frame image, simultaneously, the angle of the body key point with respect to the x-axis and the time-derivatives related to each body key point were calculated. 

The proposed method is an integrated approach consisting of body kinematics and machine learning, where it is anticipated there is a higher accuracy in fall detection. In this work, the well-known LSTM network model has been deployed. This model is concisely introduced in the upcoming sub-section.

#### 2.3.1. LSTM Model Description

As mentioned earlier, the model used in this work is LSTM network. An overview of the network architecture is shown in Figure 2. The calculated parameters, described in Section 2.3.3, are fed into the first LSTM layer and the model is trained, where its output is passed to the second layer. These two LSTM layers are followed by a dropout of 0.3 to avoid overfitting. Next, a dense layer holding one output neuron can detect falls and not-fall conditions. The Adam optimizer is used to change the attributes of the neural network, such as weights and learning rate, to reduce the losses. The first LSTM layer neurons are involved with one sequence of data. The first and second LSTM layer both hold 100 neurons. To control how well the network model learns the training dataset, the relu activation function is applied in the model. As the case in this study is a binary classification task, at last, the sigmoid activation function is used in the dense layer to distinguish between falls and not-falls. 

The concept of each layer as shown in Figure 2 is as follows:

- The input layer consists of P_1_, P_2_…, P_m_, all calculated parameters described in Section 2.3.3, where the value of m depends on the number of parameters used in the models described in Section 2.3.4.
- The hidden layers are two layers, H_1n_ and H_2n_, for the *nth* LSTM neuron in the first hidden layer, and the nth LSTM neuron in the second hidden layer, respectively.
- The output layer contains the calculated parameters mapped into two classes of fall and not-fall by a sigmoid classifier in the form of a vector. This vector has three dimensions, where it has been decomposed into a sliding window with a length of 18. The input data of LSTM are the time series of the 18 × *m* matrix.

To apply the LSTM model to the present work, we need to define an experimental space through which the coordinates of different key points of the subject’s body can be calculated. This space is defined in sub-Section 2.3.2 below.

#### 2.3.2. Visualization of the Experimental Space

To distinguish between the balance and imbalance of a human body, it is necessary to consider the body orientation with respect to a fixed coordinate system. Moreover, this coordinate system will be used to visualize the space in which the required input parameters are being calculated. Mardinez and Ponse [14] have taken the axes of the coordinate system at the outer left corner in the depth of the image, where the positive y-axis points down towards the floor, the positive x-axis points to the right side of the image, and the positive z-axis points toward the frame, as shown in Figure 1a.

To fully visualize the motion posture and also to be able to analyze the motion instability of the human body, a simplified descriptive geometry is drawn in Figure 1b. This geometry contains only three human skeleton key points by which the characteristics of the motions can be explored. This simplification takes place in the preprocessing stage with a lower complexity in the computations. It is believed that, by extracting only 3 body key points in an active image frame, and with the help of physical characteristics of a human body due to various standing postures, we might be able to predict the subject’s fall correctly. The pseudo-code of the extraction of the key points algorithm is completed through a light-weighted algorithm MediaPipe framework (Algorithm 1) below.
**Algorithm 1.** Key point detection algorithm on the human body using Media Pipe frameworkInput: The continuous frames F in the video; Output: X, Y, and Z coordinate values of key points (Kp): “Nose, Left Shoulder, Left Hip”;   for i ← 1 to #F do Capture current frame Fi; Detect the person in the current image using pose Detector (); Extract key points from the person’s body using mpPose.Pose; Calculate the key point values with respect to X-, Y-, and Z-axis coordinates using mpPose.Pose; Connect the related specified key points to form the human skeleton; Construct the human-body-specified key point set Kp;   end return Kp.

The outputs of Algorithm 1 are used to calculate some angles for the above-mentioned 3 key points (Equations (1)–(3)) below.

#### 2.3.3. Input Parameters Calculation

According to the theory of human body dynamics, the balance and stability of the human body are closely related to the position of the body and the position of the support plate (Zhang and Wu [17]). The key points that were used in this study are nose (N:1), left shoulder (LSh:2), and left hip (LH:3), as shown in Figure 1. The extracted information from images consists of only x and y-coordinates of N, LSh, and LH key points, where the other required input parameters will be calculated by these coordinates in the next steps.

The parameters used as input in the proposed models have been calculated through the following steps:

Step 1: calculation of the angle between the connected line from each body key point to the origin with the x-axis (Equation (1)):

(1) $$A_{i}=\arctan\left(\frac{Y_{i}}{X_{i}}\right)$$

where *A* is in radian, *X*, and *Y* are coordinates.

Step 2: the rate of change in angle $A_{i}$ between two consecutive frames (Equation (2));

(2) $$dw_{i}=\frac{\Delta A_{i}}{\Delta t_{i}}$$

where *i* takes *N*, *LSh*, and *LH* for nose, left shoulder, and left hip, respectively, and ∆*t* is the time interval between two consecutive frames.

Step 3: the ratio of the $N_{y}\;$to $LH_{y}$ in every active frame (Equation (3)):

(3) $$R=\frac{N_{y}}{LH_{y}}$$

In these three equations, 4 parameters of $dw_{1}$, $\;dw_{2},\;\mathrm{and}\;dw_{3}$ for nose, left shoulder, and left hip, respectively, and also *R* as the ratio of the $N_{y}\;$ to $LH_{y}$ were calculated. Here, *N_x_* and *N_y_* are the *x* and *y*-coordinates of nose, respectively.

#### 2.3.4. Models Description

Three different *Systems for Alarming of Fall in oldEr adults (SAFE)* are proposed in this work as below.

##### Model with Four Parameters (4p-SAFE)

In this model, the LSTM network will be applied and four parameters of *N_x_*, *N_y_*, $dw_{1}$ (Equation (2)), and the *R* (Equation (3)) will be used as input.

##### Model with Five Parameters (5p-SAFE)

In 5p-model, again, the LSTM network will be applied, where, this time, five parameters of *N_x_*, *N_y_*, and the 

$dw_{1},dw_{2},\;\mathrm{and}\;dw_{3}$ (Equation (2)) will be deployed as input. 

##### Model with Six Parameters (6p-SAFE)

Finally, in the 6p-model, the LSTM network with six input parameters of *N_x_*, *N_y_*, $dw_{1},$ $dw_{2},\;dw_{3}$ (Equation (2)), and the *R* (Equation (3)) will be proposed. In all three models, the network setting is as shown in Table 1.

## 3. Results and Analysis

### 3.1. Loss Function

Calculating the difference between the target value and the LSTM network’s predicted value involves using the loss function. The accuracy of the network increases with decreasing loss function. In classification issues, cross entropy, a loss function that characterizes the magnitude of the discrepancy between the model’s predicted value and the actual value, is frequently utilized. Suppose *p* is a real event, *q* is the probability of an event, and the loss function is defined as (Lin et al. [17]):

(4) $$Loss=\sum_{x=1}^{X}\sum_{i=1}^{N}-p\left(x_{i}\right)\log_{2q}\left(x_{i}\right)$$

where *N* is the size of the test set, and *X* is the classification category.

### 3.2. Model Evaluation Indices

All the models described in Section 2.3.4 are evaluated using well-known statistical parameters, as shown in Equations (5)–(8). Common evaluation criteria for classification include precision, recall, and accuracy. In order to determine the effectiveness of the classification model, the evaluation index is calculated using the above-mentioned criteria.

True (T) and false (F) represent the correct and incorrect results, respectively. True positive (TP), true negative (TN), false positive (FP), and false negative (FN) are the four categories into which these are divided. 

*Precision* is a metric that quantifies the number of correct positive predictions made. It is calculated by dividing the true positives by anything that was predicted as a positive. If the true event is non-fall (negative), the model prediction is also non-fall, and the ratio of the two is:

(5) $$Precision=\frac{1}{N}\sum_{i=1}^{N}\frac{TP_{i}}{TP_{i}+FP_{i}}\times 100\%$$

*Recall* (or true positive rate) is calculated by dividing the true positives by anything that should have been predicted as positive. If the true event is a fall (positive), the model predicts a fall as well. *Recall* is defined as the ratio of the two:

(6) $$Recall=\frac{1}{N}\sum_{i=1}^{N}\frac{TP_{i}}{TP_{i}+FN_{i}}\times 100\%$$

*F*1-*score* is a valuable measure in evaluation of binary classifications. *F*1-*score* is the weighted average of precision and recall. Therefore, this score takes both false positives and false negatives into account:

(7) $$F1-score=\frac{2\times Precision\times Recall}{Precision+Recall}\times 100\%$$

*Accuracy* refers to the proportion of the two classification targets (fall and not-fall) that are correctly judged in all classified samples. Like all events, the model correctly predicts the proportion of falls and non-falls:

(8) $$\mathrm{Accuracy}=\frac{\mathrm{TP}+\mathrm{TN}}{\mathrm{TP}+\mathrm{TN}+\;\mathrm{FP}+\mathrm{FN}}\times 100\%$$

### 3.3. Models Implementation 

#### 3.3.1. Data Pre-Processing

To effectively use these data, the following pre-processing stages have been implemented:

- Those images in which reading of three key points was not possible were not used.
- Those cases in which the subject’s hands touched the floor are assumed to show falling, and the frames afterward were not taken into account.
- The Activity IDs were labeled by the data owners (Mardinez and Ponse [14]). To achieve reliable results from machine learning tools, it is necessary to balance the data between the two classes of fall and not-fall. To use the real data only and to avoid generating extra simulated data, it was decided to apply a down-sampling operation to balance the two target classes.

Finally, 7614 data records were retained. To prepare the data as input to the machine learning model, the data were windowed according to the sampling frequency already tuned at ∼18 Hz and in a time-series order.

In this section, the three suggested models are being implemented and analyzed. As mentioned above, 7614 data values were supplied by 17 participating subjects, where 5220 of them (around 69%) were allocated to model training and the remaining 2394 data values (around 31%) were assigned to model evaluation. Out of 5220 data values, 20% were allocated to the validation process.

The deep neural network model used in this work is a long-short-term memory (LSTM) model, which is believed to solve the problem of long sequence dependence and effectively prevent gradient explosion and gradient vanishing. The most important feature of an LSTM layer is to learn temporal dependencies within sequences. Therefore, it was decided to segment the data into sequences of 18-length sliding windows (based on the frequency of data suggested by Mardinez and Ponse [14]). To preserve the temporal dependency of data, the data were not shuffled. Since the nature of LSTM networks is to keep the behavior of the data in memory as the new data sequence enters, usually, it interacts with the information already present in the memory. On the other hand, the behavior of the person should be continuously monitored, and any movement must be detected. Taking this into consideration, it is decided not to have any overlapping in the sliding windows. In this work, the window size is set to 1 s, where the window overlap is none. The average time steps were around 0.05 s. Therefore, a sliding window of a fixed size of 18 timestamps was deployed. Consequently, the columns of these windows are parameters used in the model, while the rows consist of time steps. As mentioned earlier, the total number of data is 7614 rows in 423 packs of 18 each. Out of 423 packs, 327 packs were used for model training and validations, and the remaining 96 packs were used in testing the models. 

Since the LSTM network expects 3D vector inputs, the size of each input that is being fed to the network is 1 × 18 × *P,* where *P* is the number of parameters used in each model (Figure 1).

#### 3.3.2. 4p-SAFE Model Implementation

To implement this model, the parameters mentioned in Section 2.3.4. were used as inputs. The results of the evaluation based on the criteria mentioned in Equations (5)–(8) are as follows: precision: 99.1%, recall: 97.3%, F1-score: 98.2%, accuracy: 96.99%. As can be seen, 96.99% of the cases were detected correctly. 

**Figure 3:**
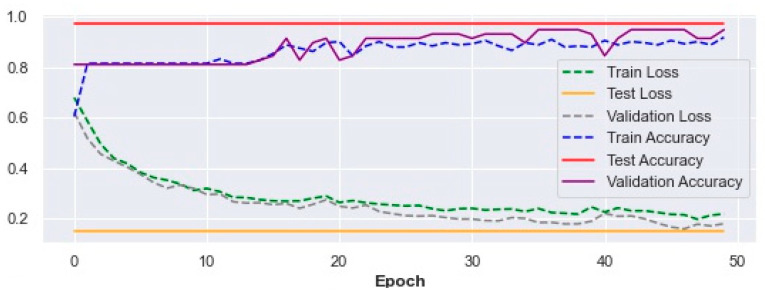
Train, test, and validation accuracy and loss results in the 4p-SAFE model.

Figure 3 shows the trend of improvement in the accuracy of training, testing, and validating and loss (Equation (4)) of fall prediction in different epochs for 4p-SAFE.

In conclusion, the 4p-SAFE model may have 3.01% error in correct detection of falls. However, we believe that the performance of this model may improve if we can feed back the detection results in the model. 

#### 3.3.3. 5p-SAFE Model Implementation

In the 5p-SAFE model, as mentioned in Section 2.3.4, the parameters *N_x_, N_y_, dw_N_*, *dw_LSh_*, and *dw_LH_* were deployed. 

By implementing the 5p-SAFE model, the results of the evaluation based on the criteria mentioned in Equations (5)–(8) are as follows: precision: 97.4%, recall: 100%, F1-score: 98.7%, accuracy: 97.74%. As can be seen, the rate of fall detection shows a noticeable improvement (97.74%) compared to 4p-SAFE (96.99%). 

**Figure 4:**
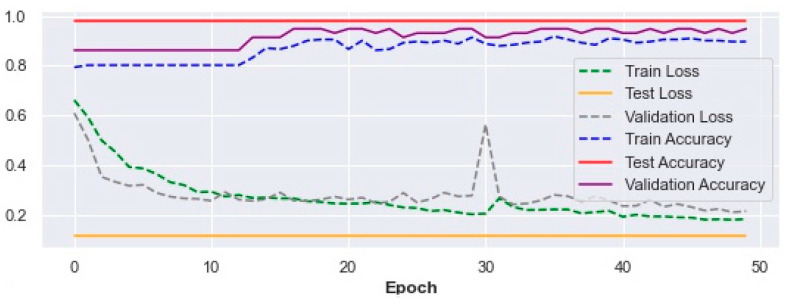
Train, test, and validation accuracy and loss results in the 5p-SAFE model.

The trend of improvement in the accuracy of fall prediction for training, testing, and validation with their corresponding loss (Equation (4)) values over 50 epochs in the 5p-SAFE are shown in Figure 4 below.

In conclusion, the 5p-SAFE model may have 2.26% error in correct detection of falls. Of course, the performance of the 5p-SAFE model may improve if more data are fed into the model and fed back. 

#### 3.3.4. 6p-SAFE Model Implementation

In this model, the parameters mentioned in Section 2.3.4 were used as inputs. The results show the correct recognition rate of falling is highest among the three suggested models. 

Using the parameters mentioned in Section 2.3.4, the results of the evaluation based on the criteria mentioned in Equations (5)–(8) are as follows: precision: 98.2%, recall: 100%, F1-score: 99.1%, accuracy: 98.5%.

According to the above results, the percentage of correct fall and not-fall diagnosis shows a significant increase, and the model performance is as high as 98.5%.

**Figure 5:**
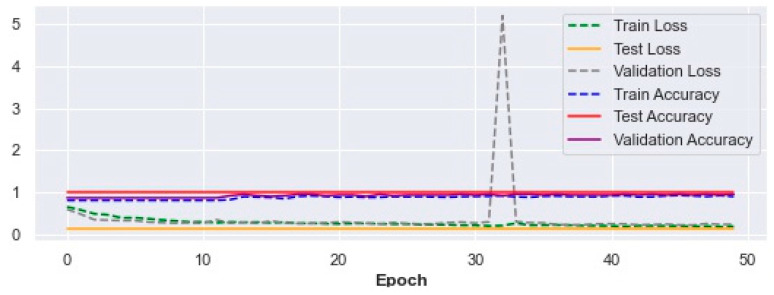
Train, test, and validation accuracy and loss results in the 6p-SAFE model.

As shown in Figure 5, the trend of improvement in the accuracy of fall prediction for training, testing, and validation with their corresponding loss (Equation (4)) values over 50 epochs in 6p-SAFE have been improved compared to 4p-SAFE and 5p-SAFE.

In conclusion, it seems that the 6p-SAFE model with six parameters as input has the ability to achieve more correct fall detection and can be deployed wherever is needed. Moreover, the performance of this model may gradually improve over time if one can feed more detection results into the model. Using each one of the aforementioned models is a tradeoff between prediction accuracy and calculation time. This means that all three models are liable to be used almost equally.

## 4. Discussion

### 4.1. 4p-SAFE

When a person is experiencing a fall, the *dw_N_* (Equation (2)) is usually higher than when he/she is bending forward intentionally. Although it seems that choosing the *dw_N_* parameter is a good choice for fall detection, there might be cases in which someone bends forward at a higher rate compared to when bending forward intentionally (for instance, in exercise). 

To be able to distinguish between balance and imbalance postures, it was decided to use *dw_N_* along with the ratio of the y-coordinate of the nose to the y-coordinate of the left hip (Equation (3)). The closer the person’s head to the ground, the larger would be this ratio. Hence, if the amount of this ratio exceeds 1 and at the same time *dw_N_* becomes large enough, the person is more likely to fall.

The final correct recognition rate of the LSTM network achieved in the 4p-SAFE model is 96.99%.

### 4.2. 5p-SAFE

When a person bends forward intentionally, even if their *dw_1_* value is large enough and the *dw_LSh_* and *dw_LH_* (Equation (2)) are not large enough, it seems it is possible for them to be able to keep their balance. On the other hand, if the *dw_LSh_* and *dw_LH_* increase steadily along with *dw_N_*, the possibility of stability would be significantly reduced and the person is more likely to fall. Therefore, it seems that using all three parameters as model input may have a significant impact regarding correct diagnosis of a fall.

The rate of fall detection shows a noticeable improvement of 97.74% in the 5p-SAFE model.

### 4.3. 6p-SAFE

The improvement in correct recognition rate in 6p-SAFE seems to be due to the deployment of the *R* (Equation (3)) parameter. There might be cases in which the observer is expecting that someone is about to fall but the person can well regain his/her stability at that moment. In these cases, we believe that the ratio *R* would help in correct fall predictions. This means that, when a person is experiencing an increase in the three parameters *dw_N_*, *dw_LSh_*, and *dw_LH,_* and at the same time their nose reaches close enough to the ground, they probably lose their balance, and a fall is almost definite. 

According to the results obtained, using the 6p-SAFE model, a significant increase in correct fall and not-fall diagnosis is achieved. The model performance shows 98.5% in accuracy.

It is found that use of body key points improves training time effectively as well as eliminating the effects of traditional image-based approaches, such as blurriness, light, and shadows (Lin et al. [18]).

The best accuracy we achieved in this work was about 98.5%, which is comparable with the work of Zhang and Wu [17], where the accuracy in fall prediction was claimed to be 97.9%. 

The difference between this work and the one of Zhang and Wu [17] lies in the technique of the Lagrangian mechanical system, which is not directly used in this work. In the work of Zhang and Wu [17], rotational energy and generalized force with five key points were used, while, in this work, only three key points and some angles that were easy to calculate with low time consumption was proposed. The assumption of the connecting rod is used in our work as well, but the change in angle of these rods and the coordinate of their endpoints is what we used in this work. The use of Lagrangian mechanics can result in some complexity because the correct mass of subjects and the density distribution throughout the body is needed in order to be able to correctly calculate the force and rotational energy where this information cannot be extracted from the image.

## 5. Conclusions

Falls, especially for older adults, are one of the common causes of injury and illness. To prevent these events and to promote the health and wellness of older adults, especially in healthcare premises and streets in daily life, accurate prediction and awareness are essential. The present study proposed an integrated approach consisting of body kinematics and machine learning. The model data consisted of video recordings collected in an experiment conducted by Mardinez and Ponse [14]. Three LSTM-network-based models of 4p-SAFE, 5p-SAFE, and 6p-SAFE were developed. The parameters needed for these models consisted of some body key points coordinates and angles extracted from the videos supplied by fixed cameras, which could be installed everywhere, especially on aged-care premises. The body key points are nose, left shoulder, and left hip. 

The results show acceptable performance in using the traditional machine learning algorithms, with acceptable accuracy of fall and not-fall recognition. These accuracy values were 96.99, 97.74, and 98.5 percent for the 4p-SAFE, 5p-SAFE, and 6p-SAFE models, respectively. In the 4p-SAFE model, four parameters that can easily be extracted from images were used. Although the accuracy in this model is lower than in the other two models, by developing a feedback mechanism, one might gradually increase accuracy in prediction. Of course, this is true for the other two models as well. However, if feedback is not possible, then the 6p-SAFE model is strongly recommended.

What makes the proposed method suitable for use in aged-care premises is highlighted as follows:

- The models have relied on minimum parameters.
- The method is novel, simple, accurate, and reliable for applying to elderlies, especially in aged-care premises.
- The method can use feedback data and improve its accuracy in prediction.

Finally, the simplicity assumed in this work expedites prediction and simplifies calculation. However, by advancements in camera technology and computer processors, more key points could be used in models of the same logic. 

## Figures and Tables

**Table 1:** General parameters of LSTM network.

Network Parameters	Values
Input Layer Nodes	100
Epochs	50
Batch size	64
Number of hidden layers	1
Dropout	0.3
Learning rate	0.001
Activation Function	Sigmoid
Optimization Algorithm	Adam
Loss Function	Binary Cross Entropy

## Data Availability

The images processed in this study are available at http://sites.google.com/up.edu.mx/har-up/ (accessed on 22 September 2020).
